# Domestic Radon Exposure and Risk of Childhood Cancer: A Prospective Census-Based Cohort Study

**DOI:** 10.1289/ehp.1306500

**Published:** 2013-08-13

**Authors:** Dimitri Hauri, Ben Spycher, Anke Huss, Frank Zimmermann, Michael Grotzer, Nicolas von der Weid, Damien Weber, Adrian Spoerri, Claudia E. Kuehni, Martin Röösli, for the Swiss National Cohort, the Swiss Paediatric Oncology Group (SPOG)

**Affiliations:** 1 Swiss Tropical and Public Health Institute, Basel, Switzerland; 2 University of Basel, Basel, Switzerland; 3 Institute of Social and Preventive Medicine, Bern, Switzerland; 4 Institute for Risk Assessment Sciences, University of Utrecht, the Netherlands; 5 Department of Radiation Oncology, University Hospital Basel, Basel, Switzerland; 6 Department of Oncology, University Children’s Hospital Zurich, Zurich, Switzerland; 7 Pediatric Hematology and Oncology, University Children’s Hospital Basel (UKBB), Basel, Switzerland; 8 Radiation Oncology Department, Geneva University Hospital, Geneva, Switzerland

## Abstract

Background: In contrast with established evidence linking high doses of ionizing radiation with childhood cancer, research on low-dose ionizing radiation and childhood cancer has produced inconsistent results.

Objective: We investigated the association between domestic radon exposure and childhood cancers, particularly leukemia and central nervous system (CNS) tumors.

Methods: We conducted a nationwide census-based cohort study including all children < 16 years of age living in Switzerland on 5 December 2000, the date of the 2000 census. Follow-up lasted until the date of diagnosis, death, emigration, a child’s 16th birthday, or 31 December 2008. Domestic radon levels were estimated for each individual home address using a model developed and validated based on approximately 45,000 measurements taken throughout Switzerland. Data were analyzed with Cox proportional hazard models adjusted for child age, child sex, birth order, parents’ socioeconomic status, environmental gamma radiation, and period effects.

Results: In total, 997 childhood cancer cases were included in the study. Compared with children exposed to a radon concentration below the median (< 77.7 Bq/m^3^), adjusted hazard ratios for children with exposure ≥ the 90th percentile (≥ 139.9 Bq/m^3^) were 0.93 (95% CI: 0.74, 1.16) for all cancers, 0.95 (95% CI: 0.63, 1.43) for all leukemias, 0.90 (95% CI: 0.56, 1.43) for acute lymphoblastic leukemia, and 1.05 (95% CI: 0.68, 1.61) for CNS tumors.

Conclusions: We did not find evidence that domestic radon exposure is associated with childhood cancer, despite relatively high radon levels in Switzerland.

Citation: Hauri D, Spycher B, Huss A, Zimmermann F, Grotzer M, von der Weid N, Weber D, Spoerri A, Kuehni C, Röösli M, for the Swiss National Cohort and the Swiss Paediatric Oncology Group (SPOG). 2013. Domestic radon exposure and risk of childhood cancer: a prospective census-based cohort study. Environ Health Perspect 121:1239–1244; http://dx.doi.org/10.1289/ehp.1306500

## Introduction

Childhood cancer is the second most common cause of death in children (after accidents) in developed countries (Jemal et al. 2010; UK Childhood Cancer Study Investigators 2000). Incidence rates of childhood malignancies increased by approximately 1% per year in Europe between 1970 and 1999 (Kaatsch et al. 2006; McKinney 2005; Steliarova-Foucher et al. 2004), and this increase did not slow down in the first 5 years after 2000 (Pritchard-Jones et al. 2006). In the United States, the incidence of childhood malignancies increased by approximately 0.5% per year between 1992 and 2007 (Kohler et al. 2011).

Low-dose ionizing radiation is hypothesized to cause childhood cancer. Radon is a decay product of uranium, a naturally occurring element in granitic and metamorphic rocks (Ball et al. 1991; Gillmore et al. 2005; Gunderson 1992). Radon emanates from soil and concentrates inside buildings. Domestic radon is a major natural source of ionizing radiation exposure. Worldwide, radon is estimated to contribute to roughly half of the average annual ionizing radiation dose (Charles 2001). In Switzerland, this figure was estimated to be 60% (Federal Office of Public Health 2011).

Because of the high fat content of red bone marrow, it has been suggested that radon gas doses delivered to this organ may be high enough to damage stem cells (Tong et al. 2012) and increase the risk of childhood leukemia (Richardson 2008). The relationship between radon exposure and childhood leukemia has been addressed in various case–control studies (Cartwright et al. 2002; Kaletsch et al. 1999; Kendall et al. 2013; Lubin et al. 1998; Maged et al. 2000; Raaschou-Nielsen et al. 2008; Steinbuch et al. 1999; Stjernfeldt et al. 1987) and ecological studies (Alexander et al. 1990; Butland et al. 1990; Collman et al. 1991; Evrard et al. 2005, 2006; Foreman et al. 1994; Gilman and Knox 1998; Henshaw et al. 1990; Lucie 1990; Muirhead et al. 1991; Richardson et al. 1995; Thorne et al. 1996a, 1996b). Most of the ecological studies reported an association between childhood leukemia and estimated domestic radon exposure. However, because these were population-level analyses, control for individual-level confounders was not possible. Results of case–control studies have been inconsistent (Laurier et al. 2001; Tong et al. 2012), with some studies reporting an association (Maged et al. 2000; Raaschou-Nielsen et al. 2008) and others not (Cartwright et al. 2002; Kaletsch et al. 1999; Kendall et al. 2013; Lubin et al. 1998; Steinbuch et al. 1999; Stjernfeldt et al. 1987). A recent analysis of a Danish case–control study reported evidence that air pollution from road traffic might enhance the association between radon and childhood leukemia (Bräuner et al. 2012). The authors speculated that attachment of radon decay products to traffic exhaust particles may have been responsible for this observation.

For central nervous system (CNS) tumors, which are almost all found in the brain (McKinney 2005), only a few ecological (Collman et al. 1991; Henshaw et al. 1990; Thorne et al. 1996b) and case–control studies (Cartwright et al. 2002; Kaletsch et al. 1999; Kendall et al. 2013; Raaschou-Nielsen et al. 2008) have been performed, also showing inconsistent results. Ecological studies have suggested an association between domestic radon concentration and CNS tumors (Collman et al. 1991; Henshaw et al. 1990; Thorne et al. 1996b). Two large case–control studies performed in Denmark (Raaschou-Nielsen et al. 2008) and the United Kingdom (Kendall et al. 2013) reported no evidence of an association. In contrast, a German study (Kaletsch et al. 1999) reported elevated risks of CNS tumors associated with radon exposures > 70 Bq/m^3^. However, the association was based on six exposed cases only.

In view of these conflicting results, we conducted a prospective census-based cohort study to investigate whether domestic radon exposure is associated with childhood cancers, particularly leukemia and CNS tumors. In addition, we evaluated whether exposure to traffic-related air pollution [i.e., nitrogen dioxide (NO_2_)] might modify associations.

## Methods

*Databases*. We used data from the Swiss National Cohort (SNC 2011), which is described in detail elsewhere (Bopp et al. 2009; Spoerri et al. 2010). Briefly, the SNC is a nationwide longitudinal research platform that links census data collected in 1990 and 2000 with birth records, mortality records, and emigration data. It includes data on all persons living in Switzerland at the time of each census, including individual- and household-level data (e.g., information on child sex, birth order within each household, and the socioeconomic status of adults based on highest education and socioprofessional category), as well as building information. Participation in the census was compulsory, and the coverage for 2000 was estimated to be 98.6% (Renaud 2004). For this study we included all children between 0 and 15 years of age living in Switzerland on 5 December 2000.

Incident cancer cases in the SNC were identified by probabilistic record linkage with the Swiss Childhood Cancer Registry (SCCR) based on birth date, sex, and residential geocodes. The SCCR is a longitudinal national database founded in 1976 by the Swiss Pediatric Oncology Group (SPOG) (Michel et al. 2008). This registry contains baseline information and long-term follow-up information on cancer patients < 21 years of age (Kuehni et al. 2012). Registration of children diagnosed with cancer before 16 years of age is estimated to be at least 95% (Kuehni et al. 2012).

**Figure 1:**
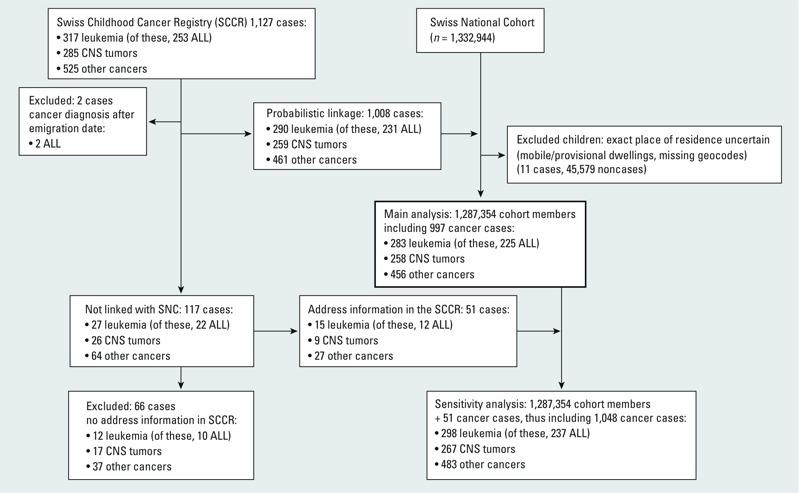
Overview on the study population obtained from linking the Swiss Childhood Cancer registry to the Swiss National Cohort. ALL, acute lymphoblastic leukemia.

Of 1,127 cases identified in the SCCR, 2 were excluded because their cancer was diagnosed after they emigrated from Switzerland and 117 were excluded because they could not be successfully linked with records in the SNC (Figure 1). The remaining 1,008 were linked to the SNC cohort consisting of 1,332,944 children. Finally, 45,590 (including 11 cases and 45,579 noncases) were excluded from our analysis because their exact place of residence was uncertain (e.g., because they were living in emergency accommodations, mobile or provisional dwellings, or buildings that could not be geocoded), leaving 997 cases and a total cohort of 1,287,354 children for the main analysis. In addition, we conducted a sensitivity analysis that also included 51 of the 117 cases who could not be linked to the SNC, but had address information from the 2000 census (1,048 cases in a total cohort of 1,287,405 children). This study is based on register data, and informed consent was not required. The SNC was approved by the ethics committees in Bern (205/06) and Zurich (13/06) and by the Federal Data Protection Office.

*Exposure assessment*. We estimated indoor radon exposure at baseline (5 December 2000) for each child’s home address using a nationwide radon prediction model (Hauri et al. 2012, 2013). The prediction model is a log-linear regression model that was developed based on 35,706 measurements, carried out in Switzerland between 1994 and 2004. Relevant predictors in the model were tectonic units, building age, building type, soil texture, degree of urbanization, and floor level (Hauri et al. 2012). The adjusted *R*^2^ was 20%. The model was validated using an independent data set of 8,925 radon measurements that were not used to develop the model. Spearman rank correlations between predicted and measured radon values were 0.45 (95% CI: 0.44, 0.46) for the development data set and 0.44 (95% CI: 0.42, 0.46) for the validation data set. Using a cut-off at the 90th percentile, areas under the ROC (receiver operating characteristic) curve were 0.73 (95% CI: 0.72, 0.74) for the development set and 0.72 (95% CI: 0.71, 0.74) for the validation set. Sensitivity was 0.31 for the development and 0.29 for the validation data set, and specificity was 0.92 for both data sets.

We evaluated potential confounders identified from the literature on environmental risk factors for childhood cancer and leukemia (McNally and Parker 2006; Tong et al. 2012). The following factors were considered: distance to major roads, railways, and electric power lines; particulate matter air pollution (PM_10_; ≤ 10 μm in aerodynamic diameter), NO_2_, and ambient benzene concentrations; exposure to radiofrequency electromagnetic fields from broadcast transmitters; and potential exposure to agricultural pesticides based on distance to the nearest orchard, vineyard, or golf course. In addition, we considered distance to the nearest pediatric cancer center because it may be associated with the completeness of childhood cancer registration, which may be better in areas with a pediatric cancer center than in the rest of Switzerland, and with the spatial distribution of radon. We estimated exposures to potential confounders from digital maps, using ArcGIS (ESRI, Redlands, CA, USA). We extracted data on background gamma radiation exposure from Swiss radiation maps (Rybach et al. 2002) with a grid cell resolution of 2 km. We obtained digital maps of power lines with a resolution of 1:25,000, from the Federal Inspectorate for Heavy Current Installations. Distances to major roads were obtained using digital maps on the traffic network with a resolution of 1:25,000 (VECTOR25-maps), published by the Federal Office of Topography (swisstopo) (2010). Data distances to orchards, vineyards, and golf courses (used to estimate exposure to agricultural pesticides) were derived from Swiss land use statistics for 1997 (Arealstatistik Schweiz), published by the Swiss Federal Statistical Office (Neuchâtel, Switzerland; http://www.bfs.admin.ch) with a grid cell resolution of 100 m × 100 m. Pediatric cancer centers were manually geocoded using the fixed point data service of the Federal Office of Topography (2010). We extracted modeled benzene levels for the year 2005 from a digital map with a grid cell resolution of 400 m, published by the Swiss Agency for the Environment, Forests and Landscape (Heldstab et al. 2004) and extracted PM_10_ and NO_2_ exposure levels from 2005 from digital maps with a grid cell resolution of 100 m, published by the Federal Office of the Environment (Heldstab et al. 2011). Exposure to analogous, digital radio, and digital TV broadcast transmitters was modeled for the year 2000 for residences within 10 km of a transmitter. Exposure to short-wave radio and medium-wave radio was modeled for the year 1997 for residences within 20 km of these transmitters. These models were developed by the Federal Office of Communications (Biel, Switzerland; http://www.bakom.admin.ch).

We used exposure to PM_10_, NO_2_, and benzene ambient concentrations as linear variables. The other factors were used categorically with predefined as exposure corridors for distance to major roads [> 400 m to highways or > 200 m to main roads (class 1), 100–400 m to highways or 50–200 m to main roads, 40–100 m to highways or 20–50 m to main roads, < 40 m to highways or < 20 m to main roads], to high voltage power lines (including railways) (< 50 m, 50–200 m, 200–600 m, > 600 m), to agricultural pesticides [distance to the nearest orchards (> 200 m, 100–200 m, 50–100 m, < 50 m)], to vineyards (> 500 m, 250–500 m, 100–250 m, < 100 m), to golf courses (> 3,000 m, 1,500–3,000 m, 750–1,500 m, < 750 m), and to the nearest pediatric center (> 30 km, 15–30 km, 5–15 km, < 5 km). Exposure categories for the radio frequency–electromagnetic frequency exposure were used, with a cut-off at 0.05 and 0.2 V/m to differentiate among low, medium, and high exposures. Residences outside the model area were considered in the lowest exposure category.

*Statistical analysis*. We analyzed data using Cox proportional hazard models with age as the underlying time scale. Time at risk began on 5 December 2005 (the date of the census) and ended on the date of diagnosis, death, emigration, the child’s 16th birthday, or 31 December 2008, whichever occurred first. We categorized exposure using *a priori* cut points at the 50th and 90th percentiles. In addition, we conducted linear exposure–response analyses of radon concentration modeled as simple continuous predictor. Hazard ratios (HRs) are expressed per 100 Bq/m^3^ increase in radon exposure. All models were adjusted for child sex, birth order within each household (linearly), socioeconomic status of the parents using the parents’ highest education (low, medium, high, no information) and their job position (low, medium, high, unemployed/retired/housewife/volunteer work, no information), as well as total background gamma radiation exposure from cosmic, terrestrial, and artificial ground radiation from the Chernobyl event [by categorizing at the 50th, 103 nSV/h (nanoSieverts per hour); and 90th percentiles, 133 nSV/h], and period effects (by dichotomizing follow-up time into two 4-year blocks). We added potential confounders to models one at a time and used a change-in-estimation criterion of 10% to select covariates for the final model (Greenland 1989). None of the potential confounders met this criterion; therefore our final models included child sex, birth order, socioeconomic status, background gamma radiation exposure, and period only. We confirmed the proportional hazard assumption using Nelson–Aalen survival functions and statistical tests based on Schoenfeld residuals and by examining variation in associations between covariates and the outcomes varied over time (data not shown).

*Subgroup and sensitivity analyses*. Because a recent case–control study (Bräuner et al. 2012) suggested an interaction between domestic radon exposure and NO_x_ (nitrogen oxides) from traffic exhaust, we stratified our analysis at the median NO_2_ concentration in our cohort (21.6 μg/m^3^). Further, we evaluated possible effect modification by sex because the risk of cancer is higher for boys than girls (Michel et al. 2008). We also conducted separate analyses for preschool children (< 5 years of age) and schoolchildren (5–15 years of age) because young children may be more vulnerable to exposure from ionizing radiation than older children (Little et al. 2010). In addition, for children 5–15 years of age, we evaluated the effect of exposure misclassification due to residential mobility (Warner et al. 1995) by conducting separate analyses of children who did or did not move residence between 1995 and 2000 based on information available in the SNC.

We also carried out a separate regional analysis for cantons that lie at least partly in the Alpine region (Grisons, Appenzell, Bern, Glarus, Lucerne, Unterwalden, Schwyz, St. Gallen, Ticino, Uri, Valais, Vaud) where the highest radon concentrations were found.

Finally, we performed a sensitivity analysis that included 51 cases who could not be linked to the SNC but had information in the SCCR on place of residence at the time of the 2000 census. Because we did not have information on the floor they lived on, building age, or building type for these children, we estimated their radon exposures assuming that they lived on the first floor of apartment buildings built between 1946 and 1970, consistent with average values for all children based on the 2000 census. These models were adjusted for sex, environmental gamma radiation, and period effects, but not for socioeconomic status of the parents or birth order.

## Results

In the SNC database 1,332,944 children were identified who were between 0–15 years of age on the date of the 2000 census. Of these, 45,590 were excluded because their exact place of residence was unclear (Figure 1). In total, we analyzed data from 1,287,354 children, accumulating 7,627,646 person-years during the study period. From the 1,127 cancer cases identified in the SCCR who were diagnosed between 2000 and 2008, 997 could be linked to the SNC database. Of these, 283 were diagnosed with leukemia [including 225 with acute lymphoblastic leukemia (ALL)] and 258 with a CNS tumor.

The estimated median radon concentration for all cohort members was 77.7 Bq/m^3^, and the 90th percentile was 139.9 Bq/m^3^ (see Supplemental Material, Table S1). The arithmetic mean radon concentration was 85.7 Bq/m^3^ (range, 6.9–337.2 Bq/m^3^) for childhood cancer cases and 85.9 Bq/m^3^ (range, 0.7–490.1 Bq/m^3^) for the rest of the study population. Arithmetic mean radon concentrations were lowest (84.0 Bq/m^3^) for ALL cases and highest for CNS tumor cases (88.9 Bq/m^3^).

**Figure 2:**
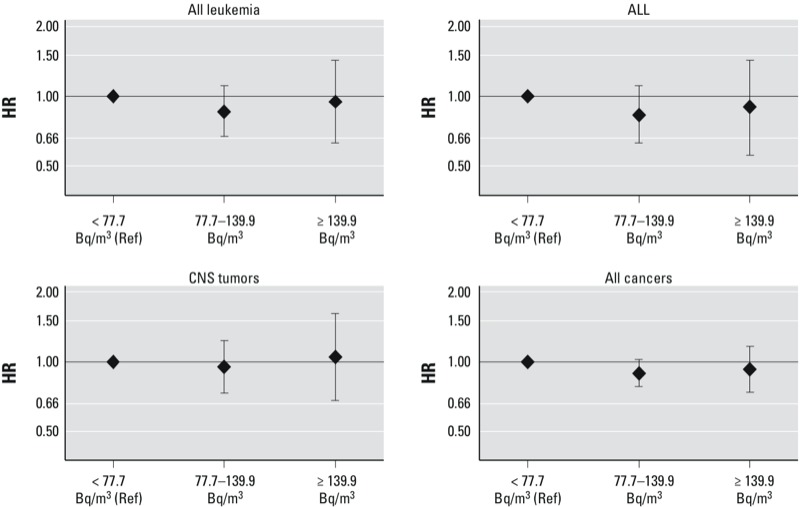
HRs and 95% CIs for associations between domestic radon concentrations at baseline and all leukemias, ALL, CNS tumors, and all cancers diagnosed among Swiss children during 2000–2008. Ref, reference. Models are adjusted for child sex, birth order, socioeconomic status of the parents, environmental gamma radiation, and period effects, in addition to using age as the underlying time scale.

Results of the main analysis are shown in Table 1 and Figure 2. Compared with children exposed to a radon concentration below the median, HRs for children with exposure ≥ 90th percentile (≥ 139.9 Bq/m^3^) were 0.93 (95% CI: 0.74, 1.16) for all cancers, 0.95 (95% CI: 0.63, 1.43) for all leukemias, 0.90 (95% CI: 0.56, 1.43) for ALL, and 1.05 (95% CI: 0.68, 1.61) for CNS tumors. Age-adjusted risk estimates were very similar to the fully adjusted results (Table 1). There was no evidence of linear exposure–response associations for any of the outcomes (Table 1). Including 51 additional cancer cases who had address information but could not be linked to the SNC had little influence on effect estimates (see Supplemental Material, Table S2). The subgroup analyses also did not indicate evidence of effect modification by age, sex, or moving status (see Supplemental Material, Table S3). Restricting the analyses to Alpine cantons, where radon levels are highest, also did not indicate an association between domestic radon concentration and childhood cancer (data not shown). Analyses stratified according to low or high NO_2_ exposure (< 21.6 or ≥ 21.6 μg/m^3^, respectively) did not provide evidence of an interaction between NO_2_ and domestic radon concentration for any of the outcomes (Table 2).

**Table 1:** Age-adjusted and fully adjusted hazard ratios (HRs) for childhood cancer and residential radon exposure.

Cancer type	Radon exposure	No. of cancer cases	Person-years	Age-adjusted HR (95% CI)	Fully adjusted HR (95% CI)^*a*^
All cancers	EGPhE	525	3,838,101	Reference	Reference
	77.7–139.9 Bq/m^3^	373	3,034,923	0.90 (0.79, 1.03)	0.89 (0.78, 1.02)
	≥ 139.9 Bq/m^3^	99	754,623	0.96 (0.78, 1.19)	0.93 (0.74, 1.16)
	per 100 Bq/m^3^	997		1.01 (0.87, 1.16)	0.99 (0.85, 1.14)
All leukemias	EGPhE	149	3,838,101	Reference	Reference
	77.7–139.9 Bq/m^3^	104	3,034,923	0.90 (0.70, 1.15)	0.86 (0.67, 1.11)
	≥ 139.9 Bq/m^3^	30	754,623	1.04 (0.70, 1.54)	0.95 (0.63, 1.43)
	per 100 Bq/m^3^	283		0.97 (0.74, 1.27)	0.90 (0.68, 1.19)
ALL	EGPhE	121	3,838,101	Reference	Reference
	77.7–139.9 Bq/m^3^	81	3,034,923	0.86 (0.65, 1.15)	0.83 (0.63, 1.11)
	≥ 139.9 Bq/m^3^	23	754,623	0.99 (0.63, 1.55)	0.90 (0.56, 1.43)
	per 100 Bq/m^3^	225		0.94 (0.69, 1.28)	0.86 (0.63, 1.19)
CNS tumors	EGPhE	132	3,838,101	Reference	Reference
	77.7–139.9 Bq/m^3^	99	3,034,923	0.95 (0.73, 1.23)	0.95 (0.73, 1.23)
	≥ 139.9 Bq/m^3^	27	754,623	1.05 (0.69, 1.59)	1.05 (0.68, 1.61)
	per 100 Bq/m^3^	258		1.18 (0.91, 1.54)	1.19 (0.91, 1.57)
For the categorical analysis, radon exposure levels were categorized at 50th and 90th percentile of the exposure ­distribution. ^***a***^In addition to using age as the underlying time scale, adjusted for child sex, birth order, socioeconomic status of the parents, environmental gamma radiation, and period effects.					

**Table 2:** Age-adjusted and fully adjusted hazard ratios (HRs) for childhood cancer and radon exposure within strata of NO_2_ concentration.

Cancer type and NO_2_ exposure all cancers	Radon exposure	No. of cases	No. of person-years	HR (95% CI)^*a*^
All cancers
NO_2_ < 21.6 μg/m^3^	EGPhE	220	1,690,638	Reference
	77.7–139.9 Bq/m^3^	185	1,635,275	0.85 (0.70, 1.03)
	≥ 139.9 Bq/m^3^	70	465,612	1.08 (0.82, 1.43)
NO_2_ ≥ 21.6 μg/m^3^	EGPhE	305	2,147,462	Reference
	77.7–139.9 Bq/m^3^	188	1,399,648	0.96 (0.80, 1.15)
	≥ 139.9 Bq/m^3^	29	289,011	0.74 (0.50, 1.11)
All leukemias
NO_2_ < 21.6 μg/m^3^	EGPhE	63	1,690,638	Reference
	77.7–139.9 Bq/m^3^	44	1,635,275	0.69 (0.47, 1.02)
	≥ 139.9 Bq/m^3^	22	465,612	1.08 (0.65, 1.80)
NO_2_ ≥ 21.6 μg/m^3^	EGPhE	86	2,147,462	Reference
	77.7–139.9 Bq/m^3^	60	1,399,648	1.07 (0.77, 1.49)
	≥ 139.9 Bq/m^3^	8	289,011	0.77 (0.37, 1.62)
ALL
NO_2_ < 21.6 μg/m^3^	EGPhE	54	1,690,638	Reference
	77.7–139.9 Bq/m^3^	34	1,635,275	0.62 (0.40, 0.95)
	≥ 139.9 Bq/m^3^	17	465,612	0.92 (0.52, 1.64)
NO_2_ ≥ 21.6 μg/m^3^	EGPhE	67	2,147,462	Reference
	77.7–139.9 Bq/m^3^	47	1,399,648	1.08 (0.75, 1.58)
	≥ 139.9 Bq/m^3^	6	289,011	0.78 (0.33, 1.82)
CNS tumors
NO_2_ < 21.6 μg/m^3^	EGPhE	60	1,690,638	Reference
	77.7–139.9 Bq/m^3^	49	1,635,275	0.86 (0.59, 1.26)
	≥ 139.9 Bq/m^3^	18	465,612	1.14 (0.66, 1.96)
NO_2_ ≥ 21.6 μg/m^3^	EGPhE	72	2,147,462	Reference
	77.7–139.9 Bq/m^3^	50	1,399,648	1.05 (0.73, 1.52)
	≥ 139.9 Bq/m^3^	9	289,011	0.91 (0.44, 1.89)
For the categorical analysis, radon exposure levels categorized at 50th and 90th percentile of the exposure distribution. NO_2_ exposure levels categorized at 50th percentile of the exposure distribution. ^***a***^In addition to using age as the underlying time scale, adjusted for child sex, birth order, socioeconomic status of the parents, environmental gamma radiation, and period effects.				

## Discussion

Our census-based cohort study did not indicate an association between domestic radon concentration and childhood cancer. The results were consistent across various sensitivity and subgroup analyses, and for different types of cancer.

To our knowledge, other cohort studies on domestic radon concentration and childhood cancers have not been published. The main strength of the present study is its nationwide coverage, which substantially reduces the likelihood of selection bias. Exposure assessment was based on a comprehensive prediction model that was developed and validated using > 40,000 measurements taken throughout Switzerland between 1994 and 2004. Previous case–control studies have reported participation < 55%, and exposure measurements were often limited to subsets of study participants (Cartwright et al. 2002; Kaletsch et al. 1999; Lubin et al. 1998; Maged et al. 2000; Steinbuch et al. 1999; Stjernfeldt et al. 1987). In contrast with ecological studies, we had information on a number of potential individual-level confounders (Laurier et al. 2001; Tong et al. 2012), although adjusting for these variables did not materially affect hazard ratios, suggesting little or no confounding by these factors although we cannot completely exclude residual confounding due to misclassification in the confounder variables. This is consistent with the current knowledge on childhood cancer etiology: There is evidence of increased risks among children with a genetic predisposition and among those exposed to high doses of ionizing radiation (e.g., applied for cancer treatment), but little evidence of environmental risk factors (Belson et al. 2007; Eden 2010; McKinney 2005; Pollack and Jakacki 2011). Only two previous case–control studies had similar methodological features to the present study—large sample size, consideration of confounding, radon exposure estimation based on prediction models, and a small likelihood of selection bias due to the use of population-based controls identified from registries without requiring consent for participation (Kendall et al. 2013; Raaschou-Nielsen et al. 2008). In contrast with our study, a Danish study reported that domestic radon exposure was associated with ALL (rate ratio = 1.56; 95% CI: 1.05, 2.30 per 1,000 Bq/m^3^–years) based on 860 cases diagnosed between 1968 and 1994, and 1,720 registry-based controls (Raaschou-Nielsen et al. 2008). However, no association was reported between radon concentrations and CNS tumors (rate ratio = 0.92; 95% CI: 0.69, 1.22 per 1,000 Bq/m^3^–years based on 922 CNS tumor cases). In a British study, the estimated relative risk for leukemia per 1,000 Bq/m^3^–years increase in cumulative radon exposure was 1.12 (95% CI: 0.88, 1.43) based on 9,058 cases and 11,912 controls, and the corresponding estimate for CNS tumors was 1.15 (95% CI: 0.88, 1.50) based on 6,585 cases and 8,997 controls (Kendall et al. 2013).

Recently, associations between radon and nonrespiratory cancers also have been investigated in adults. Consistent associations were not observed between nonrespiratory cancer mortality and ecologic measures of residential radon levels in the large prospective American Cancer Society cohort, which includes > 1 million participants (Turner et al. 2012). For example, the HR for leukemia mortality was 0.93 (95% CI: 0.82, 1.05) per 100-Bq/m^3^ increase in mean county-level residential radon concentrations. These findings are consistent with a collaborative analysis of 11 studies of miners that indicated that leukemia mortality was not associated with radon exposure (Darby et al. 1995). Wheeler et al. (2012) reported evidence of an association between radon levels and skin cancer in an ecological study conducted in southwest England during 2000–2004 (Wheeler et al. 2012). The authors speculated that radon and its decay products are attracted to water molecules, and that the resulting aerosols could adhere to the skin via electrostatic attraction. Such a mechanism was also proposed in a subsequent analysis of the Danish case–control study that reported evidence that air pollution (NO_x_) from road traffic strengthened associations between radon and childhood leukemia (Bräuner et al. 2012). Our study results, however, do not support such an interaction.

Our study also has limitations, and given the fact that we did not observe an association the main concern may be that we have missed a true association due to lack of power, or exposure misclassification. Our study included fewer cases than did the two large register-based case–control studies from Denmark (Raaschou-Nielsen et al. 2008) and Great Britain (Kendall et al. 2013). However, estimated exposure levels were larger in our Swiss study population on average (arithmetic mean radon concentration, 86 Bq/m^3^; range, 0.7–490.1 Bq/m^3^) than in the Danish (arithmetic mean concentration, 48 Bq/m^3^; range, 4 to 254 Bq/m^3^) and British studies (arithmetic mean radon in the control group, 21.3 Bq/m^3^; range, 1.2–692 Bq/m^3^). Little et al. (2010) pointed out that in epidemiological studies of cancer and ionizing radiation, statistical power is influenced much more by differences in mean dose than by the number of cases. Thus, in terms of statistical power, the large differences in exposure levels of our study population may at least partly compensate for the smaller number of cases. Regarding exposure misclassification, we deal in our study mainly with a Berkson-type error because we used a prediction model (Heid et al. 2004; Raaschou-Nielsen et al. 2008; Steenland et al. 2000). Unlike errors of individual measurements, this type of error does not bias estimates of associations towards unity, but instead reduces statistical power resulting in wider confidence intervals (Armstrong 1998; Steenland et al. 2000). Although non-Berkson error may have been introduced in the exposure assessment if people changed their place of residence, associations based on cohort members who did not relocate during the 5 years before 2000 were similar to estimates for the cohort as whole, suggesting that exposure misclassification did not substantially bias our findings.

The observed lack of an association between domestic radon exposure and childhood leukemia or CNS tumors is consistent with expectations, given low estimated doses of exposure to domestic radon for red bone marrow and the CNS. For a 1-year-old child, an annual radon concentration of 100 Bq/m^3^ [i.e., the radon concentration where remedial actions are recommended according to the World Health Organization (2009)] corresponds to an equivalent dose to the lung of 19.6 mSv per year (Kendall and Smith 2005). Organ-specific doses for red bone marrow (0.43 mSv) or the brain (0.19 mSv) are much smaller (Kendall and Smith 2002, 2005). Comparable values were estimated for 10-year-old children (lung: 21.1 mSv; red bone marrow: 0.52 mSv; and brain: 0.14 mSv) (Kendall and Smith 2005). These dose estimations support our observed results and suggest that doses from domestic radon levels to organs other than the lung are too weak to noticeably increase cancer risks.

## Conclusions

In summary, we did not find evidence that domestic radon exposure is associated with childhood leukemia or CNS tumors, despite relative high radon levels in Switzerland.

## Correction

The values for age-adjusted hazard ratios (HRs) (95% CIs) for all leukemias in Table 1 and for HR (95% CI) for all leukemias (NO_2_ < 21.6 μg/m^3^, ≥ 139.9 Bq/m^3^) in Table 2 were incorrect in the manuscript originally published online. They have been corrected here.

## Supplemental Material

(332 KB) PDF
