# Supplementary material for: Domestic Radon Exposure and Risk of Childhood Cancer: A Prospective Census-Based Cohort Study
Source: Environ Health Perspect. 2013 Aug 13;121(10):1239–44. doi: 10.1289/ehp.1306500 (PMC3801468; doi:10.1289/ehp.1306500)
Supplement: (332 KB) PDF [file ehp.1306500.s001.pdf]

Supplemental Material

Domestic Radon Exposure and Risk of Childhood Cancer: A Prospective Census-Based Cohort Study

Dimitri Hauri, Ben Spycher, Anke Huss, Frank Zimmermann, Michael Grotzer, Nicolas von der Weid, Damien Weber, Adrian Spoerri, Claudia Kuehni, and Martin Röösli for the Swiss National Cohort and the Swiss Paediatric Oncology Group (SPOG)

Table of Contents

Supplemental Material, Table S1: Characteristics of all cohort members................................p 2

Supplemental Material, Table S2: Consideration of additional 51 cancer cases not linked to the SNC but with addresses available at the date of census 2000 (December 5 2000)...................p 3

Supplemental Material, Table S3: Subgroup analyses of the association between domestic radon levels and various childhood cancer types by gender, age-group and history of moving..........p 4

Supplemental Material, Table S1: Characteristics of all cohort members.

Characteristics		Study participants No. (%)	Cancer cases [No. (%)]				Radon exposure of all cohort members (Bq/m³)		
			All cancers	Leukemia	ALL	CNS	arithmetic mean	median	90th percentile
All participants		1,287,354 (100)	997 (100)	283 (28.4)	225 (22.6)	258 (25.9)	85.9	77.7	139.9
Gender	boys	658,960 (51.2)	561 (56.3)	168 (59.4)	133 (59.1)	138 (53.5)	85.9	77.7	139.9
	girls	628,394 (48.8)	436 (43.7)	115 (40.6)	92 (40.9)	120 (46.5)	85.9	77.7	139.7
Age	< 5 years	373,017 (29.0)	170 (39.0)	72 (25.4)	62 (27.6)	42 (16.3)	84.3	76.1	137.6
	5-15 years	914,337 (71.0)	609 (61.0)	211 (74.6)	163 (72.4)	216 (83.7)	86.6	78.5	140.2
Parents' socio-economic status									
Education	low	165,126 (13.4)	106 (11.1)	23 (8.3)	20 (9.1)	27 (10.8)	80.4	72.0	134.9
	medium	627,851 (50.8)	476 (49.6)	153 (55.4)	116 (52.5)	117 (45.446.6)	88.5	79.9	143.8
	high	443,770 (35.9)	377 (39.3)	100 (36.2)	85 (38.5)	107 (42.6)	85.4	78.2	136.0
	no information[a]	50,607	38	7	4	7	74.6	67.0	122.8
Job position	low	131,441 (11.1)	89 (9.7)	20 (7.5)	16 (7.6)	19 (8.1)	83.6	74.6	140.2
	medium	487,787 (41.2)	393 (43.0)	120 (44.9)	98 (46.5)	107 (45.7)	86.2	78.2	138.5
	high	233,786 (19.8)	193 (21.1)	52 (19.5)	42 (19.9)	51 (21.8)	84.6	77.4	134.0
	unemployed/retired/ housewife/volunteer work	330,609 (27.9)	239 (26.2)	75 (28.1)	55 (26.1)	57 (24.4)	88.8	80.0	144.6
	no information[a]	103,731	83	16	14	24	81.4	73.3	133.3

Age at time of census 2000 was considered for indicating the number of all cohort members and cancer cases and when reporting radon concentrations (arithmetic mean, median, 90th percentile. Age at time of diagnosis was considered when indicating the number of cancer cases.

[a]Education, job position: observations with missing data were included within a separate category in the analyses.

Supplemental Material, Table S2: Consideration of additional 51 cancer cases not linked to the SNC but with addresses available at the date of census 2000 (December 5 2000).

Cancer type	Radon exposure	No. cancer cases	No. of person-years	Crude HR (95% CI)	Adjusted HR (95% CI)
All cancers (n=1,048)	<77.7 Bq/m³	558	3,838,207	Reference	Reference
	77.7-139.9 Bq/m³	384	3,034,959	0.90 (0.79, 1.03)	0.89 (0.78, 1.02)
	≥ 139.9 Bq/m³	106	754,648	0.96 (0.78, 1.19)	0.93 (0.74, 1.16)
All leukaemias (n=298)	<77.7 Bq/m³	158	3,838,207	Reference	Reference
	77.7-139.9 Bq/m³	109	3,034,959	0.89 (0.69, 1.13)	0.88 (0.69, 1.12)
	≥ 139.9 Bq/m³	31	754,648	1.02 (0.69, 1.49)	0.97 (0.65, 1.45)
ALL (n=236)	<77.7 Bq/m³	127	3,838,207	Reference	Reference
	77.7-139.9 Bq/m³	86	3,034,959	0.86 (0.65, 1.15)	0.86 (0.65, 1.13)
	≥ 139.9 Bq/m³	24	754,648	0.99 (0.63, 1.55)	0.92 (0.58, 1.47)
CNS tumors (n=267)	<77.7 Bq/m³	135	3,838,207	Reference	Reference
	77.7-139.9 Bq/m³	103	3,034,959	0.96 (0.75, 1.25)	0.96 (0.74, 1.24)
	≥ 139.9 Bq/m³	29	754,648	1.10 (0.74, 1.65)	1.09 (0.72, 1.65)

Abbreviations: ALL, acute lymphoblastic leukemia; CI, confidence interval; CNS tumors, central nervous system tumors; HR, hazard ratio

fully adjusted models**:** in addition to using age as the underlying time scale, adjusted for gender, the environmental gamma radiation and period effects. Radon exposure levels categorized at 50th and 90th percentile of the exposure distribution.

Supplemental Material, Table S3: Subgroup analyses of the association between domestic radon levels and various childhood cancer types by gender, age-group and history of moving.

Cancer type	Radon exposure	Gender				Age				Moving status between 1995 and 2000: 5-15 years old			
		boys		girls		< 5 years		5-15 years		not moved		moved	
		No. cases	adj. HR (95% CI)	No. cases	adj. HR (95% CI)	No. cases	adj. HR (95% CI)	No. cases	adj. HR (95% CI)	No. cases	adj. HR (95% CI)	No. cases	adj. HR (95% CI)
All cancers	<77.7 Bq/m³	285	Reference	240	Reference	87	Reference	438	Reference	192	Reference	110	Reference
	77.7-139.9 Bq/m³	218	0.95 (0.80, 1.14)	155	0.82 (0.67, 1.00)	65	0.99 (0.72, 1.38)	308	0.87 (0.75, 1.01)	144	0.83 (0.67, 1.03)	72	0.85 (0.63, 1.15)
	≥ 139.9 Bq/m³	58	1.00 (0.74, 1.34)	41	0.85 (0.60, 1.20)	18	1.09 (0.64, 1.85)	81	0.90 (0.70, 1.15)	44	0.87 (0.62, 1.23)	16	0.85 (0.50, 1.46)
All leukaemias	<77.7 Bq/m³	84	Reference	65	Reference	38	Reference	111	Reference	40	Reference	25	Reference
	77.7-139.9 Bq/m³	67	0.99 (0.71, 1.36)	37	0.70 (0.47, 1.05)	28	0.95 (0.58, 1.56)	76	0.82 (0.61, 1.10)	31	0.83 (0.52, 1.34)	20	1.00 (0.55, 1.80)
	≥ 139.9 Bq/m³	17	0.95 (0.55, 1.63)	13	0.97 (0.52, 1.80)	6	0.77 (0.32, 1.90)	24	1.00 (0.63, 1.58)	12	1.02 (0.52, 2.03)	5	1.22 (0.46, 3.24)
ALL	<77.7 Bq/m³	66	Reference	55	Reference	33	Reference	88	Reference	29	Reference	20	Reference
	77.7-139.9 Bq/m³	53	0.99 (0.69, 1.43)	28	0.64 (0.40, 1.01)	23	0.93 (0.54, 1.59)	58	0.80 (0.57, 1.11)	21	0.76 (0.43, 1.35)	15	0.93 (0.48, 1.82)
	≥ 139.9 Bq/m³	14	0.94 (0.51, 1.72)	9	0.85 (0.41, 1.75)	6	0.92 (0.37, 2.28)	17	0.88 (0.51, 1.51)	6	0.66 (0.26, 1.68)	5	1.43 (0.52, 3.95)
CNS tumors	<77.7 Bq/m³	70	Reference	62	Reference	19	Reference	113	Reference	50	Reference	27	Reference
	77.7-139.9 Bq/m³	51	0.89 (0.62, 1.29)	48	1.01 (0.69, 1.48)	19	1.35 (0.71, 2.58)	80	0.88 (0.66, 1.18)	36	0.80 (0.52, 1.23)	16	0.75 (0.40, 1.39)
	≥ 139.9 Bq/m³	17	1.19 (0.68, 2.08)	10	0.87 (0.44, 1.73)	4	1.37 (0.46, 4.11)	23	0.99 (0.62, 1.59)	12	0.96 (0.50, 1.85)	3	0.54 (0.16, 1.88)

Abbreviations: ALL, acute lymphoblastic leukemia; CI, confidence interval; CNS tumors, central nervous system tumors; HR, hazard ratio. Fully adjusted models**:** in addition to using age as the underlying time scale, adjusted for gender, birth order, socio-economic status of the parents, the environmental gamma radiation and period effects. Radon exposure levels categorized at 50th and 90th percentile of the exposure distribution.
